# Controlling the Microbiome: Microhabitat Adjustments for Successful Biocontrol Strategies in Soil and Human Gut

**DOI:** 10.3389/fmicb.2016.01079

**Published:** 2016-07-13

**Authors:** Eveline Adam, Anneloes E. Groenenboom, Viola Kurm, Magdalena Rajewska, Ruth Schmidt, Olaf Tyc, Simone Weidner, Gabriele Berg, Wietse de Boer, Joana Falcão Salles

**Affiliations:** ^1^Institute of Environmental Biotechnology, Graz University of TechnologyGraz, Austria; ^2^Laboratory of Genetics, Wageningen UniversityWageningen, Netherlands; ^3^Department of Terrestrial Ecology, Netherlands Institute of Ecology, The Royal Netherlands Academy of Arts and SciencesWageningen, Netherlands; ^4^Laboratory of Biological Plant Protection, Intercollegiate Faculty of Biotechnology, University of Gdańsk and Medical University of GdańskGdańsk, Poland; ^5^Department of Microbial Ecology, Netherlands Institute of Ecology, The Royal Netherlands Academy of Arts and SciencesWageningen, Netherlands; ^6^Department of Soil Quality, Wageningen University and Research CentreWageningen, Netherlands; ^7^Department of Biology, Institute of Environmental Biology, Utrecht UniversityUtrecht, Netherlands; ^8^Institute of Evolutionary Life sciences, Groningen UniversityGroningen, Netherlands

**Keywords:** host beneficial bacteria, microbiome control, minor disturbances, major disturbances, synbiotics

## Introduction

The human gut and the rhizosphere are environments colonized by highly diverse communities of microbes, which perform complex interactions with their host and carry out important functions including enhanced disease resistance and nutrient uptake. In humans they are involved in energy harvest and storage as well as in interactions with the immune system (Clemente et al., 2012). In plants they have profound effects on seed germination, seedling vigor, nutrition, plant health, and development of the innate immune system (Mendes et al., 2013; Berg et al., 2014a; Schikora et al., 2016). The composition of the microbial communities is host-specific and related to its health status (Smalla et al., 2001; Kinross et al., 2011; Berg et al., 2014a). Imbalances caused by disturbance-induced shifts in microbial species abundances can lead to disease outbreaks in both environments (Berendsen et al., 2012; Robles Alonso and Guarner, 2013; Berg et al., 2014b) and further to probable proliferation of pathogenic species (Van Elsas et al., 2012; Van Agtmaal et al., 2015).

To restore or maintain the health of the host, bio-based solutions supporting the pathogen-suppressing ability of the hosts' native microbiome can be applied, including probiotics, synbiotics and biocontrol agents (de Vrese and Schrezenmeir, 2008). Such methods aim to increase the abundance and activity of host beneficial bacteria (HBB). However, addition of HBB does not always result in the desired pathogen suppression due to insufficient establishment, i.e., lower survival and/or poor colonization rates of the HBB (Bashan et al., 2014).

Concepts from invasion ecology suggest that survival rates of invaders are inversely related to the diversity of the native microbiome. This can be explained by higher resource uptake and consequent reduction in niche availability (Mallon et al., 2015). In addition, prevailing physical and chemical parameters in the respective environment like texture, pore size distribution, and moisture content might not favor the establishment of the introduced HBB. For a long-term establishment of the HBB in the soil these abiotic factors have to be considered. In the gut, the colonization resistance determined by the commensal microbiome is linked to its capacity to exploit the available niches and to prevent the establishment of invaders via niche occupation (reviewed in Stecher et al., 2013). The knowledge on mechanisms of microbial invasions (Mallon et al., 2015) can be used to improve the survival of HBB in both environments.

Given that similar mechanisms drive microbial colonization and establishment in the gut and rhizosphere microbiomes, we suggest that biocontrol strategies could be similar for both environments (Ramírez-Puebla et al., 2013; Berg et al., 2015; Mendes and Raaijmakers, 2015). Here we develop possible strategies to ensure long-term establishment of HBB by manipulating niche availability.

## Creating microhabitats for host beneficial bacteria by introducing minor disturbances

Several studies have shown that soils harboring low microbial biomass or low microbial diversity are more susceptible to colonization by other organisms (Fließbach et al., 2009; Van Elsas et al., 2012). Certain agricultural practices can result in major disturbances of the **rhizosphere microbiome**. Examples include disinfestation with chemical pesticides, heat treatment (Stapleton, 2000), radiation or anaerobic disinfestation (Van Agtmaal et al., 2015). Moreover, tillage systems may have major effects on the established community by reducing certain soil microbial populations, particularly fungi (Ventorino et al., 2012). Analogous events, leading to changes in the **human gut microbiome**, are the application of broad spectrum antibiotics, fecal transplantations (Landy et al., 2011; de Vos, 2013) or considerable changes in diet (Turnbaugh et al., 2009). Whilst major disturbances are frequently used to eliminate pathogens, those methods possibly also disrupt beneficial functions of the indigenous microbial community (Altieri, 1999; Geiger et al., 2010).

An alternative strategy is to introduce **minor disturbances** to create free niches for HBB's in both the rhizosphere and the human gut microbiome. This strategy aims to selectively empty niches in the existing community.

In the rhizosphere the introduction of accessory bacterial predators such as protozoa (e.g., flagellates, ciliates) or nematodes (Jousset et al., 2006; Abada et al., 2009; Pedersen et al., 2009; Freyth et al., 2010; Neidig et al., 2011; Müller et al., 2013) could foster biocontrol strains via enhanced selective predation when the biocontrol strain protects itself through production of antibiotics. The increase in predation pressure might also stimulate biocontrol strategies by direct predation on pathogens as well as nutrient turnover and bacterial activity in soil. Likewise, specific bacteriophages could be applied to selectively eliminate target bacterial species or strains. This strategy has been effectively shown as part of disease management for *Rhizobium* sp., *Bacillus* sp., *Burkholderia* sp., *Xanthomonas* sp., *Pectobacterium* sp. and *Dickeya* sp. (Evans et al., 1979; Sharp et al., 1986; Lynch et al., 2012; Chae et al., 2014; Santamaría et al., 2014; Czajkowski et al., 2015). For this approach, elimination of pathogens and reduction of soil bacterial species that directly compete with the biocontrol agents (i.e., those sharing similar metabolic capacities) are desirable. Due to their specificity, bacteriophages have also been used to treat gastrointestinal infections of bacterial origin in humans (Sulakvelidze et al., 2001; Abedon et al., 2011). Moreover, they were successfully used together with bifidobacteria to treat antibiotic-associated dysbacteriosis in infants (Litvinova et al., 1978). Therefore, bacteriophages represent an alternative to selectively wipe out bacteria (either pathogens or strong competitors) in the gut and to form a niche for potential HBBs to thrive. In the rhizosphere the use of bacterial helper strains, an application of targeted specific antibiotics or enzymes (e.g., chitinases; Herrera-Estrella and Chet, 1999) might affect the microbiome composition sufficiently to form free niches for HBB. Another possibility is to introduce minor changes in physical properties like pH value (Rousk et al., 2010), temperature (Van Veen et al., 1997; Haas and Défago, 2005), moisture dynamics or salinity (Canfora et al., 2014; Dini-Andreote et al., 2014).

Most of the methods described here apply to the rhizosphere (e.g., substantial temperature or salinity changes), but due to ethical concerns can only be considered in a limited manner for the human gut. Thus, the direct applicability to the human gut remains to be investigated. The concept of freeing/forming a microhabitat for HBB by minor disturbances in the rhizosphere or the human gut should be developed and optimized for different situations.

Apart from making an existing niche available for the HBB by removal of at least a part of the adapted community, creation of a new niche could also be taken into consideration.

## Improvement of the environment—the human gut as a paragon for concepts in biocontrol

To alleviate competition and increase the chance of establishment of HBB in an environment that harbors a highly diverse microbial community utilizing all available resources can be enabled by adding specific energy resources, for example prebiotics. Prebiotics selectively stimulate growth and/or activity of the beneficial bacteria and facilitate their establishment in the heavily colonized gut (Teitelbaum and Walker, 2002; Tuohy et al., 2003). Moreover, administration of synbiotics, a combination of a probiotic (i.e., the HBB) and a prebiotic, has recently attracted attention (Schrezenmeir and de Vrese, 2001). The prebiotic provides a selective food source for the HBB enhancing its growth and establishment (Teitelbaum and Walker, 2002; Saulnier et al., 2008). The success of synbiotics has been demonstrated *in vitro* as well as *in vivo* (Bartosch et al., 2005; Saulnier et al., 2008). We suggest that the use of synbiotics in the human gut can serve as a paragon to enhance the establishment of HBB in the soil. In the rhizosphere the addition of a selective food source e.g., rhizopins (Oger et al., 2004) could be used to stimulate specific bacteria in the rhizosphere community.

## Synbiotics for the soil

Parallels with prebiotics can be seen in the application of general resources to the soil, such as composts and green manures. These strategies have shown to be effective in the control of soil-borne diseases as they combine the introduction of biocontrol microorganisms with organic matter after the thermophilic phase low in competition and free nutrients. This substrate favors the growth of beneficial microbes and suppresses the growth of saprophytic pathogens (Hoitink et al., 1997; Hoitink and Boehm, 1999). A disadvantage of using this method, however, is varying compost quality, which results in inconsistent colonization by biocontrol agents and subsequent effects on disease-suppression (Sturz and Christie, 2003). To ensure the presence of the desired HBB, composts can be fortified with specific beneficial microorganisms or amended with substrates that stimulate growth and activity of a selected group of microorganisms (Haggag and Abo-Sedera, 2005; Chae et al., 2006; Dukare et al., 2011).

In addition, specific substrates and HBB can be combined to complement each other. Several studies have shown that certain carbon sources and minerals increase the activity of biocontrol bacteria (Duffy and Défago, 1999; Shaukat and Siddiqui, 2003; Kim et al., 2008). Moreover, plants are able to select for specific bacteria by exudation of sugars, polysaccharides, amino acids, and a variety of secondary metabolites (Teplitski et al., 2000; Badri et al., 2009). These compounds are comparable to mucosal glycans in the human gut. As a soil synbiotic, these compounds could be artificially applied in combination with the respective HBB. Not only nutrient sources, but also signaling molecules and chemo-attractants should be taken into account, which are often highly specific for certain bacterial species or even strains. Ultimately, engineering beneficial microbes or genetically modified plants that are capable of synthesizing certain enzymes quenching bacterial signal particles might allow for shaping microbial communities against plant host pathogens (Dong et al., 2001; Ryan et al., 2009).

To support a long shelf life and stability of the product, these compounds can be formulated with specific carrier materials, membrane stabilizers and buffering agents in fine-tuned quantities (Paau, 1998; Bashan et al., 2014). An example for such soil inoculum carrier is biochar (charcoal used as soil amendment), known to have positive effects on soil properties such as pH (Saxena et al., 2013; Hale et al., 2014) and potentially be amended with extra HBB-specific resources.

## Utilizing the specificity of host-bacterium interactions

Selection of the appropriate crop plant or a particular bacterial genotype can significantly influence the growth and establishment of HBB in soil (Mazzola, 2004) as interactions between plant and bacterial genotypes are assumed to be highly specific. This specificity could also be used in the human gut hosting defined beneficial strains (Tap et al., 2009). This selection could counteract down-sides of synbiotics, in which the presence of the HBB usually decreases dramatically once the consumption of the prebiotic stops (Bezkorovainy, 2001). For the same reasons, enhancing indigenous soil bacteria should be considered as an alternative to introducing new strains as they are likely to be better adapted to the respective environment (Chaparro et al., 2012).

## Outlook and conclusions

**Figure 1:**
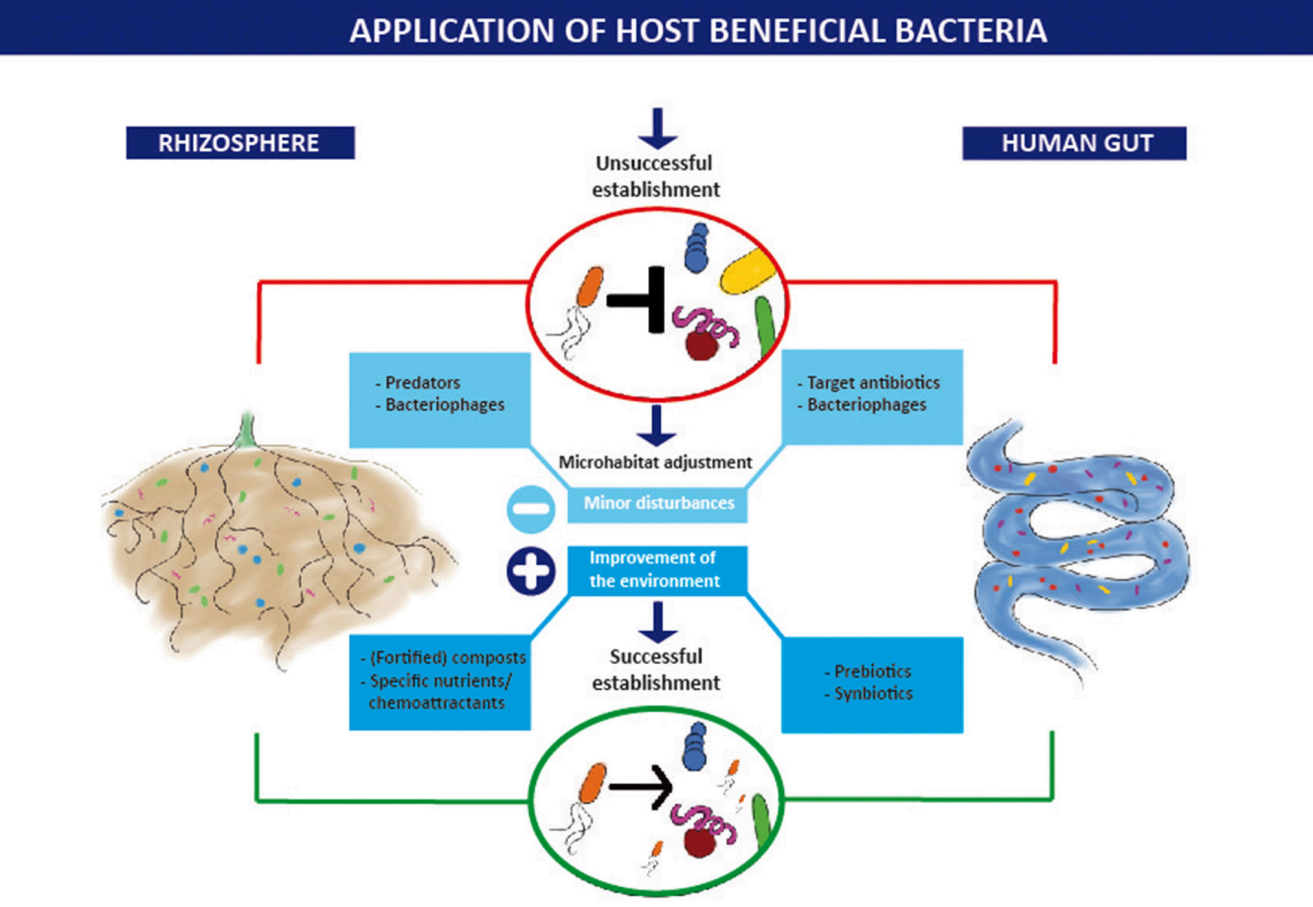
**Process of establishment of host beneficial bacteria (HBB) in the rhizosphere soil and the human gut**. Application of HBB without additional measures frequently results in an unsuccessful establishment. To achieve a successful establishment of the HBB introduction of minor disturbances that empty host-associated niches in combination with improvements of the environment that create a new niche are suggested. Examples for the rhizosphere and the human gut.

In our opinion, the future of the HBB application lies in milder treatment of soils by using case-specific nutrient-microbe combinations as well as individualized treatments of patchy field sites after field structure analysis. As summarized in Figure 1, we suggest considering approaches such as the use of minor disturbance combined with timely application of HBB to improve their establishment in the soil. Soil treatments could be selected in analogy to therapies chosen for human guts. A new term “**synbiotics for the rhizosphere**” could reflect such intention.

It is assumed, that modern crop plants lost beneficial traits due to breeding programs conducted under conditions with high nutrient supply and the use of chemical pesticides. Consequently, breeding plants for beneficial plant-microbe interactions is an emerging research topic that might give birth to cultivars, which interact more efficiently with beneficial indigenous strains or with the applied HBB.

We see a sustainable future for agriculture by comparing methods for restoring or retaining the human gut microbiome and those altering the rhizosphere microbiome. Therefore, we suggest a paradigm shift in agricultural practices toward specialized treatment of the rhizosphere microbiomes as described in this work. We invite researchers of agricultural and human health related research areas to compare the methods of both fields and take into consideration findings of the other for their own future work.

## Author contributions

The authors EA, AG, VK, MR, RS, OT, and SW contributed equally to this opinion paper. GB, WD, and JF shared senior contribution.

### Conflict of interest statement

The authors declare that the research was conducted in the absence of any commercial or financial relationships that could be construed as a potential conflict of interest.
